# Reducing urinary oxalate by simultaneous using Sankol herbal drop with oxalate-degrading bacteria

**Published:** 2019-12

**Authors:** Rouhi Afkari, Mohammad Bokaeian, Soroosh Dabiri, Habib Ghaznavi, Mohsen Taheri, Fereshteh Heidari Tajabadi, Mohammad Mehdi Feizabadi

**Affiliations:** 1 Department of Microbiology, School of Medicine, Infectious Diseases and Tropical Medicine Research Center, Resistant Tuberculosis Institute, Zahedan University of Medical Sciences, Zahedan, Iran; 2 Department of Laboratory Sciences, Zahedan University of Medical Sciences, Zahedan, Iran; 3 Department of Pharmacology, School of Medicine, Cellular and Molecular Research Center, Zahedan University of Medical Sciences, Zahedan, Iran; 4 Department of Genetics, School of Medicine, Genetics of Non-Communicable Disease Research Center, Zahedan University of Medical Sciences, Zahedan, Iran; 5 Department of Microbiology, School of Medicine, Tehran University of Medical Sciences, Tehran, Iran

**Keywords:** Herbal medicine, Hyperoxaluria, Oxalate degrading bacteria, Probiotic bacteria, Sankol drop

## Abstract

**Background and Objectives:**

Oxalate degrading bacteria and herbal extracts are new strategy for reducing hyperoxaluria. In Iranian traditional medicine, Sankol oral drop is widely used as an antispasmodic drug to reduce stones from urinary tract. This study aimed to evaluate the synergistic effect of oxalate-degrading bacteria and Sankol oral drop in reducing urinary oxalate in rat model.

**Materials and Methods:**

Several bacterial strains, including *Lactobacillus* (4), *Bifidobacterium* (2) and *L. paracasei* (2) (very strong in degrading oxalate *in vitro*) were used in this study. Male Wistar rats were divided into 6 groups (n = 6). The rats of Group I received normal diet and drinking water + 60% ethanol (positive group). Groups II (negative group), III, IV, V, and VI rats received diet containing ethylene glycol (3%) for 30 days. Groups III rats received Sankol with minimum concentration (7.5 ml/kg/b.w), Group IV rats received Sankol with maximum concentration (9 ml/kg/b.w), Group V rats received Sankol with minimum concentration + probiotic, and Group VI rats received Sankol with maximum concentration + probiotic for 30 days.

**Results:**

Treatment with Sankol (maximum concentration) and oxalate-degrading probiotic bacteria significantly reduced urinary oxalate (*P* = .0001). At the end of treatment period, rats in groups II (negative control) showed a high score of CaOx crystal, while rats in VI groups did not show any CaOx crystal.

**Conclusion:**

This is the first study on the simultaneous use of Sankol herbal drop and oxalate-degrading probiotic bacteria that showed a significant reduction in urinary oxalate.

## INTRODUCTION

Urolithiasis, stone formation in the urinary tract, including kidneys, is an important health problem experienced by many people (1). Nowadays, due to the sucessive effect of herbal remedies on the treatment of various diseases, their use is increasing (2). There are four types of kidney stones: calcium, cysteine, struvite, and uric acid (3). Approximately 80% of stone forming individuals form calcium oxalate stones (4). Hyperoxaluria is a risk factor for kidney stones, and the urine load of oxalate plays a pivotal role in calcium oxalate stone formation even in normocalciuric patients (5). Oxalate is derived from endogenous metabolic sources, mainly produced by the liver, but also by dietary absorption of oxalate that can contribute as much as 50% of what is in urine (6). The etiology of this disorder is multifactorial and is strongly related to dietary lifestyle habits (7). Increased rates of hypertension and obesity, which are linked to nephrolithiasis, also contribute to increased stone formation. On the other hand, scientific researches have validated many traditional remedies. The use of ethnomedical information has also contributed to worldwide health care by identifying bioactive compounds for direct use in medicine (8). Sankol oral drop is one of the plants that are widely used in Iranian traditional medicine. Sankol oral drop contains extracts of *Foeniculum vulgare, Laurus nobilis, Tribulus terrestris, Cuminum cyminum, Cucumis melo, Zea mays* and *Cerasus avium,* which are widely used as an antispasmodic to reduce urinary tract stones (9, 10). The active ingredients of medicinal plants in Sankol drop directly relax the smooth muscles of the urinary tract and, with their diuretic activity, remove the kidney stones, with up to 7 mm in diameter, and facilitate the expelling of urolith from kidney. Sankol drop has a potent anti-cholinergic activity, and thus alleviates the spasm and colic pain of urinary tracts due to stone presence in kidney (11). It has also been documented that gut commensal bacteria with oxalate-degrading activity have the potential to contribute to oxalate homeostasis (6). Maintaining a normal ecology among the bacterial species that constitute the endogenous digestive microflora is a natural defense mechanism against urolithiasis (6, 7). The commonly described intestinal bacteria known to degrade oxalate are categorized into 2 groups: (I) the “generalist oxalotrophs”, including some strains of *Bifidobacterium* and *Lactobacillus genera*, which degrade alternative carbon sources in addition to oxalate; and (II) the “specialist oxalotrophs”, such as *Oxalobacter formigenes*, which is a commensal anaerobe that uses only oxalate as its sole carbon source (5–8). A potential role for these bacteria in contributing to oxalate homeostasis is a decrease in the intraluminal oxalate load available for absorption across the intestine because of this oxalate-degrading action. Therefore, one of the objectives of the present study was to evaluate the anti-hyperoxaluria activity of Sankol, an herbal medicine and probiotics, in a rat model.

## MATERIALS AND METHODS

### Experimental animals.

Male Wistar rats weighing 200–250 g were used in the study. They were housed in a laboratory and kept at 12 hours light– dark cycle, controlled room temperature (23 ± 2ºC), and relative humidity (50 ± 10%). Also, they were given a standard diet and drinking water and exposed to 0.3% of ethylene glycol with 1% ammonium chloride in their drinking water for 3 days. Later, 0.3% ethylene glycol in drinking water was continued for 30 days. The rats (test groups) were then exposed to Sankol oral drop (purchased from Goldaru. Co. Iran) at doses of 7.5 (minimum concentration) and 9 (maximum concentration) (ml/kg/body weight), respectively.

### Grouping of rats.

Male Wistar rats were divided into 6 groups (n=6). Group I received normal diet and drinking water + 60% ethanol (positive control group). Groups II (negative control group), III, IV, V, and VI rats received 3% ethylene glycol containing diet for 30 days. Group III rats received Sankol with minimum concentration (7.5 ml/kg/b.w), Group IV rats received Sankol with maximum concentration (9 ml/kg/b.w), Group V rats received Sankol with minimum concentration + probiotics, and Group VI rats received Sankol with maximum concentration+ probiotic for 30 days.

### Preparation of probiotic culture.

Bacterial strains used in the study were as follow: *Lactobacillus acidophilus* PTCC 1643, *Lactobacillus Delbrukii* PTCC 1737, *Lactobacillus plantarum* PTCC 1745, *Lactobacillus casei* PTCC 1608, *Bifidobacterium bifidum* PTCC 1644, *Bifidobacterium animalis* subsp. *lactis* PTCC 1736, *Streptococcus salivarius* subsp. *thermophilus* PTCC 1738, *L. paracasei AKPL-IR (JF461540.1),* and *L. paracasei AKKL-IR (JF461539.1)*. To prepare the bacteria, 1g of lyophilized bacteria was inoculated into 100 mL of MRS broth medium (Merck, Germany) and the inoculated media was incubated in microaerophilic condition. One ml of medium was transferred into 99 mL of the new MRS broth medium and diluted to 1%. Then, it was placed at 37°C for 6 to 8 hours. The cultures were transferred to the fresh medium during the week for the number of cells needed. Finally, they were kept in a refrigerator at 4°C. The probiotic cells were isolated after centrifugation at 2422g at 4°C for 10 minutes. Then, the separated cells were washed twice using the solution of 0.1% v/v peptone.

### Determining oxalate-degrading capability of bacterial strains.

All cultures were incubated in aerobic and anaerobic condition at 37°C for 48 hours. Base media containing ammonium oxalate was prepared to determine the growth of each strain and its dependent on oxalate, as the energy source comparing the base media lacked ammonium oxalate. Finally, all selected-strains were cultured in an ammonium oxalate plate (20 mM, 40 mM and 60 mM) to assess their oxalate degrading capability (Table 3).

### Survival in a low pH environment.

Probiotic strains were evaluated for their resistance under low pH environment. Bacterial cells were harvested from overnight cultures, washed twice in phosphate buffer (pH 6.5), and suspended in the MRS broth adjusted with 1 N HCl to pH 2.5. The cells were incubated anaerobically at 37°C and their survival rate was measured at intervals of 0, 30, 60, 120, 180, and 240 minutes using the plate count method (Table 3).

### Resistance to bile salts.

Resistance to bile was examined using MRS agar plates supplemented with 0.5%, 1.0%, and 5.0% (w/v) bile (Oxgall; 70168 sigma). The probiotic bacteria were inoculated into MRS broth and incubated at 37°C under anaerobic condition for 24 hours. Strains were spot inoculated (10 μL) onto the various concentrations of bile plates and incubated at 37°C under anaerobic condition for 48 hours. The growth rates on porcine bile plates were compared to the growth rate on MRS agar plates (Table 3).

### Collecting urine and serum samples.

On 0, 15^th^, and 30^th^ days of the study period the rats were placed in metabolic cages and 24-hour urine samples were collected in tubes containing sodium azide (0.02%v/v) to prevent bacterial growth. The specimens were aliquoted for various tests after determining their volume and pH. Urinary oxalate, calcium, and creatinine were assayed using the commercial kit (Darman Keve, Isfahan, Iran) in semi-automatic photometer according to manufacturer’s protocol. Each week, 1-hour urine samples were collected before the start of 24 hours urine sample collection and were examined by light microscopy to analyze CaOx-crystalluria. The rats were anesthetized with Xylasein/Ketamine by intramuscular (IM) injection (Xylasein 0.55 and Ketamine 10 mg/kg Body Weight) and blood was taken from orbital sinus into the centrifuge tube without anticoagulant, allowed to clot at room temperature, and centrifuged to collect serum. Sera were tested for creatinine and blood urea nitrogen (BUN).

### *In vivo* urinary oxalate levels using selected pro-biotic.

Probiotics (10^11^ CFU/ml) prepared in distilled water were given to the rats (intervention group) for 4 weeks (6 animals in each group). Rats were weighed weekly and urine samples were collected on weeks 0, 2, and 3, and 4 by placing the animals in metabolic cages for 24 hours.

### Analysis of histopathology and CaOx crystal in kidney.

In this study, the presence of CaOx crystal in each kidney tissue was examined by pizzolato staining method. At first, the kidney tissue was fixed in 10% neutral buffered formalin, trimmed, processed, and set in paraffin. Sections from each kidney were stained with hematoxylin and eosin and examined under the light microscope for pathological analysis and polarized light microscope for visualizing CaOx crystal. The presence of CaOx crystal was scored on a basis of CaOx.

### Statistical analysis.

The statistical analysis of between different groups was performed based on one-way and two-way ANOVA using SPSS version 16 and MSTACT software. Statistical significance was set at *p*<0.05.

## RESULTS

### Co-treatment of Sankol drop and probiotic improved primary health of hyperoxaluric rats.

In this study, rats in positive control group (Group I) received standard chow and rats in other groups (Groups II, III, IV, V, VI) received mixed food stayed. Experimental rats that stone formation were induced in them by ethylene glycol remained healthy and put on weight. However, over the time, experimental rats significantly gained less weight than the positive control ones (*p*<0.05), while rats in groups V and VI receiving the co-treatment of Sankol drop with probiotic gained more weight than the negative control group (Group II). In experimental rats, pH of urine was lower than the negative control group. Also, pH of groups V and VI was lower than groups III and IV, which was similar to urine pH of rats in the positive control group. The parameters are displayed in Tables 1 and 2. Urine oxalate, creatinine, and calcium were decreased significantly (*p*=0.001) in groups V and VI as compared with groups III and IV (Tables 1 and 2).

**Table 1.** Urinary Oxalate, Calcium and Creatinine levels in positive and negative control groups in -30 days

**Group**		**Oxalate (mm/day)**	**Creatinine (mg/day)**	**Calcium (mg/day)**
Positive control	Mean	0.3767	825 . 333	1.3267E2
	Std. Deviation	0.04967	52.68649	6.24233
Negative control	Mean	1.6267	1484.0000	4.9333E2
	Std. Deviation	0.28994	16.13691	1.03473E1

All values are expressed as Mean, Std. Deviation (n=6) animals in each group.

**Table 2.** Urinary Oxalate, Calcium and Creatinine levels in Co-treatment group (sankol with probiotic bacteria) in 30 days

**Group**		**Oxalate (mm/day)**	**Creatinine (mg/day)**	**Calcium (mg/day)**
Sankol (maximum concentration)	Mean	0.5850	1102.5000	1.1650E2
	Std. Deviation	0.11167	50.98137	6.89202
Sankol (maximum concentration) with probiotic	Mean	0.3117	768.1667	1.7250E2
	Std. Deviation	0.11444	50.48729	5.12835
Sankol (minimum concentration)	Mean	0.8683	1220.0000	2.8167E2
	Std. Deviation	0.12968	98.99495	7.25718
Sankol (minimum concentration) with probiotic	Mean	0.40200	1122.1667	2.5800E2
	Std. Deviation	0.13286	35.22168	5.86515

**Fig. 1.**
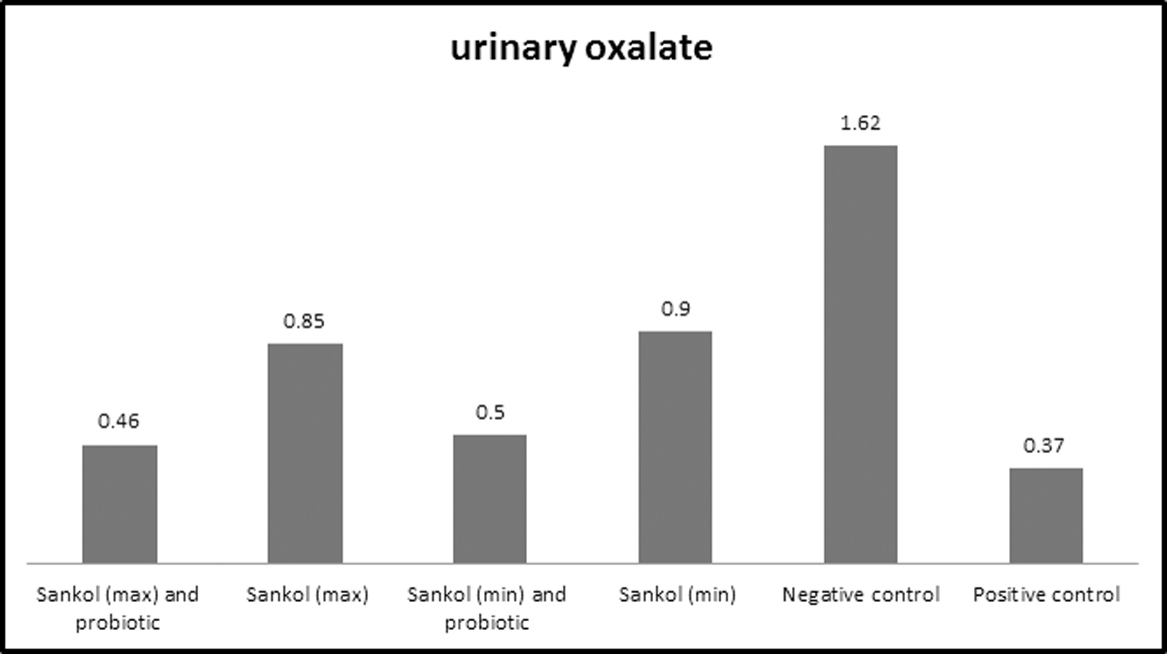
Urinary oxalate levels in all studied groups after 30 days. The Max and Min words are concentrations of sankol.

Excretion of urinary oxalate showed a significant increase in rats of negative control group (*P*<0.05), but it decreased in groups V and VI at the end of the experiment (30 days) compared to groups III and IV (Fig. 1).

The excretion of urinary calcium increased in the negative control group, but it was significantly lower in experimental group (*P*<0.05). Also, comparison of urine creatinine in each group showed that (simultaneous use of Sankol drop with probiotic) the decrease of creatinine was significant at day 30 only in the experimental group.

Also, serum BUN and creatinine were decreased in groups V and VI as compared to rats in negative control group (Group II). On the other hand, in the rats co-treated with Sankol and probiotic (Groups V and VI), the level of creatinine decreased compared to the creatinine levels in rats of negative control group and the groups that consumed either bacteria or Sankol. All values are expressed as mean ± SEM.

### Prevention of crystalluria in Sankol-probiotic treated rats.

**Fig. 2.**
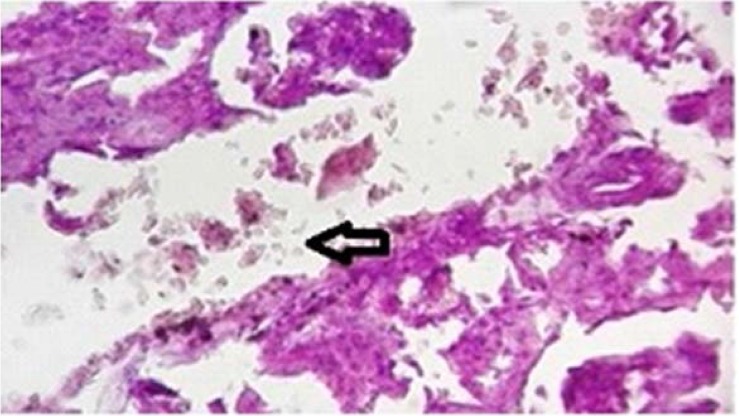
Accumulation of calcium oxalate crystals and tissue necrosis in kidney tubules in the negative control group (Groups II). As can be seen in the figure, the accumulation of calcium oxalate crystals has resulted in tissue damage.

**Fig. 3.**
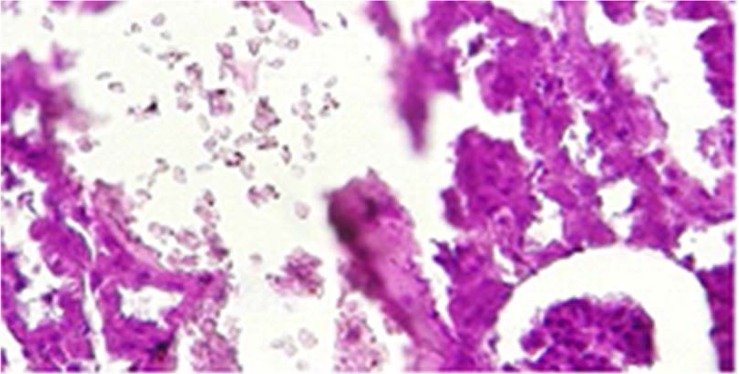
Accumulation of calcium oxalate crystals in kidney tubules in group use of Sankol (maximum concentration of Sankol dropwithout probiotic) (Groups IV).

**Fig. 4.**
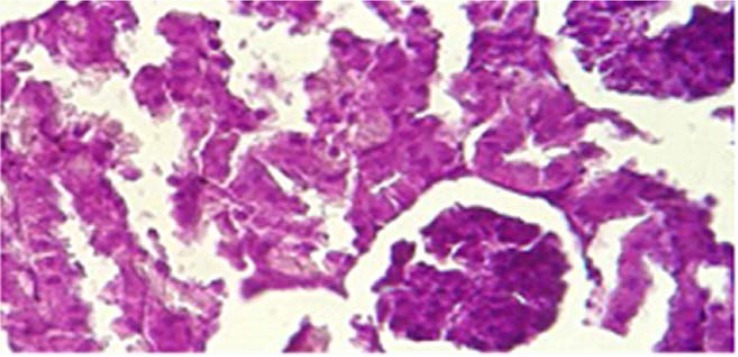
Accumulation of calcium oxalate crystals in kidney tubules in the co-treatment group (maximum concentration of Sankol drop with probiotic) (Groups VI).

All experimental rats were examined for the presence of CaOx crystal in kidney after the administration of ethylene glycol, Sankol drop, and probiotics. In Group I (positive control rats), kidney was devoid of any CaOx crystal throughout the experimental period. By day 30, rats in Group II (negative control rats) showed high score of CaOx crystal, while in group IV (Sankol with maximum concentration) kidney showed low score, but in Group V and, especially VI Group, rats did not show any CaOx crystal (Figs 2–4).

Also, all strains tested showed resistance to gastric pH (2.5-2.0) and bile salts (0.3%) (Table 3).

**Table 3.** Charactrestic of all strains used in this study

**Strains**	**Medium**	**Culture Condition**	**oxalate-degrading capability**	**Sensitivity to gastric pH**	**Resistance to bile salts (0.3%)**
*Lactobacillus acidophilus* PTCC 1643	MRS Medium with cysteine	37°C, microaerophilic, anaerobic	+	+	+
*Lactobacillus casei* subsp. *casei* PTCC 1608	MRS broth Medium	37°C microaerophilic, anaerobic	+	+	+
*Lactobacillus delbrueckii* subsp. *bulgaricus* PTCC 1737	MRS broth Medium	37°C, microaerophilic, anaerobic	+	+	+
*Lactobacillus plantarum* subsp. *plantarum* PTCC 1745	MRS broth Medium	37°C, 5% CO_2_ microaerophilic, anaerobic	+	+	+
*Bifidobacterium* animalis subsp. *lactis* PTCC 1736	Bifidobacterium Medium	37°C, anaerobic	+	+	+
*Bifidobacterium bifidum* PTCC 1644	Bifidobacterium Medium	37°C, anaerobic	+	+	+
S*treptococcus salivarius* subsp. *thermophiles* PTCC 1738	Trypticase Soy Yeast Extract Medium	37°C, microaerophilic	+	+	+

## DISCUSSION

Kidney stone formation is one of the oldest human health problems. The prevalence of kidney stone is high in the US (8%) followed by Japan (7%), Germany (5%), and Korea (4%) (11–14). In Iran, the prevalence of kidney stone differs according to geographical location, with the highest frequency in southeast (9.6%) and southern (8.5%) provinces and the lowest frequency in the northern provinces (1.4%) (12–14). Diet, high salt intake, urinary tract infection, overuse of antibiotics, and genetic status have been reported as the most important risk factors affecting the prevalence of kidney stones in Iran (12–14). In 2016, the prevalence of calcium oxalate stones reported as 61.25% (12–16). Researchers claim that there is a direct relationship between the oxalate calcium stone formation and use of oxalate-rich foods (spinach, almond, cashews, grits, beets, cocoa powder, okra); also use of many common antibiotics that reduce the oxalate-degrading bacteria has been reported (16, 17). The oxalate degrading bacteria live in the digestive tract and cause the oxalate to be balanced (18–20). Laboratory studies indicate that most mammals are not able to decompose oxalate and, therefore, excrete it through urine, which ultimately causes calcium oxalate deposition and kidney stone formation (19, 21). Recently, various researches have demonstrated the use of oxalate-degrading bacteria in various *in vitro* and *in vivo* experiments for the treatment of hyperoxaluria (5, 15, 24). However, extensive research suggests that probiotic bacteria have high oxalate degradation potential. Oxalate-degrading bacteria convert oxalate to formate and carbon dioxide by certain enzymatic pathways (22, 23).

Besides, the interest of modern medicine in herbal medicine has increased, and traditional therapies have suggested as new therapies to improve various diseases, including kidney stones (11–13). Several *in vitro* studies focused on calcium oxalate crystallization in the presence or absence of a particular plant extract. In 2010, Hossein et al., found that *Cucumismelo, Carica papaya* and *Pinus eldarica* could block crystal binding to cultured renal cells (25).

Similar effects on calcium oxalate crystallization *in vitro* have been shown for an aqueous extract from *Foeniculum vulgare*, a plant used for the treatment of stone disease (26, 27).

This study was conducted to evaluate the effect of the simultaneous use of oxalate-degrading bacteria and Sankol drop in reducing urinary oxalate in rat model. In this study, Sankol herbal drop and oxalate degrading bacteria were used. Each 30 ml of Sankol drop contains extract of *Foeniculum vulgare* 25%, *Laurus nobilis* 12.5%, *Tribulus terrestris* 12.5%, *Cuminum cyminum* 12.5%, *Cucumis melo* 12.5%, *Zea mays* 12.5%, *Cerasus avium* 12.5%. Their chemical elements and ingredients include: *Zea mays stigmata*: tannins, allantoin, bitter glycosides, cryptoxanthin, phytosterols, car vacrol, menthol, thymol, flavonoids; *Foeniculum vulgare*: anethole, fenchone andestragole (methylchavicol); *Tribulus terrestris*: glycosides, tribuloside, astragallin, alkaloids harmane and harmine; *Cucumis melo*: melonin, some trypsin –inhibitors; *Laurus nobilis*: lauric acid, cineol; *Cuminum cyminum*: cuminaldehyde, gamma terpenes, beta pinenes, parecymene (26–29). The pharmacological actions of Sankol are approved in traditional medicine. Sankol drop directly relaxes the smooth muscles of urinary tract (28) and due to its diuretic activity, removes kidney stones, and facilitates the expel of kidney precipitations. Sankol drop has a very potent anticholinergic activity and thus alleviates the spasm and colic pain in the urinary tract (29) due to the presence of stones. Mechanisms of action of Sankol drop, include the anti-urolithiasis activity of an ethanolic extract of the fruits of Tribulus terrestris in rats which were artificially affected by induced urolithiasis. It was revealed that the effect of Sankol drop dose-dependent and completely inhibited stone formation. *Tribulus terrestris* extract showed both diuretic and antimicrobial activities (28). The antimicrobial activity of Sankol drop is due to its essential oils mostly anethole and fenchone (29, 30). All of the chemical compounds mentioned in the herbal drop of Sankol, are responsible analgesic, anti-inflammatory, antispasmodic effects, and removal of kidney and urinary tract stones (30). In this study, at first, a week before the start of the experiment, the formation of kidney stone was induced in rats using ethylene glycol.

The period of recovery, treatment and reduce the level of their urine oxalate, or calcium oxalate sediments, complications and inflammation of the kidney tissue was much longer, while with using oxalate degrading bacteria with Sankol herbal drop, the period treatment and recovery and removal of calcium oxalate stones were much faster than using Sankol herbal drop without oxalate degrading bacteria.

Also, in this study, Probiotic strains were evaluated for their resistance to low pH and bile salts, the samples of *Lactobacillus* spp. were evaluated for their tolerance or not to the gastric pH and bile salts as a way to measure the potential of these probiotics, that all strains showed resistance to gastric pH (2.0) and bile salts (0.3%). The results of both studies of Durban and Scher, found a low sensitivity to 0.3% bile salts (32, 31). However, the results of such studies have found high tolerance of strains to gastric pH, which was observed in this work.

It was indicated that oxalate degrading probiotic bacteria are very effective in reducing inflammation and eliminate of calcium oxalate stones, rather than using herbal medicines alone (without oxalate degrading bacteria) to remove kidney stones. Also, there was a significant relationship between use of probiotic bacteria and reduced level of urinary oxalate.

## CONCLUSION

Increasing urinary oxalate and frequent calcium oxalate crystallization cause inflammation and degeneration of the renal tissue, lead to clinical problems and high economical load. According to the results of the present study, Sankol herbal drop and oxalate degrading bacteria has synergistic effect in decreasing inflammation of the renal tissue and levels of urinary oxalate *in vivo* test. We hope that use of probiotic bacteria with herbal medicines could provide a new perspective on the treatment of kidney stones.
